# ScFv T1 Protects Against Mitochondrial Damage of SH-SY5Y Cells Caused by Extracellular Tau Aggregates

**DOI:** 10.3390/antiox15040515

**Published:** 2026-04-21

**Authors:** Zongbao Wang, Xinyi Jiang, Jingye Lin, Ruiheng An, Yulian He, Sen Li

**Affiliations:** Gene Engineering and Biotechnology Beijing Key Laboratory, The Key Laboratory of Cell Proliferation and Regulation Biology of Ministry of Education, College of Life Sciences, Beijing Normal University, Beijing 100875, China; 202231200029@mail.bnu.edu.cn (Z.W.); 202421200034@mail.bnu.edu.cn (X.J.); 202131200026@mail.bnu.edu.cn (J.L.); 202321200007@mail.bnu.edu.cn (R.A.); 202321200012@mail.bnu.edu.cn (Y.H.)

**Keywords:** mitochondrial damage and dysfunction, extracellular Tau aggregates, oxidative stress, neuronal death, single-chain variable fragment antibody

## Abstract

Mitochondria are essential organelles that perform irreplaceable functions in neurons. The degeneration of neurons in Alzheimer’s disease (AD) is associated with mitochondrial damage, and Tau pathology represents a significant pathogenic factor in AD. However, the relationship between Tau and mitochondrial dysfunction during neuronal degeneration remains unclear. In this study, we investigated the effects and mechanisms by which extracellular Tau aggregates induce neuronal mitochondrial damage and dysfunction. The results showed that extracellular Tau aggregates lead to structural damage of mitochondria in SH-SY5Y cells and disrupt mitochondrial homeostasis. Extracellular Tau aggregates can also cause mitochondrial oxidative stress and inhibit oxidative phosphorylation in SH-SY5Y cells. Concurrently, extracellular Tau aggregates promote neuronal death through an increase in cytochrome C, mtDNA leakage and activation of the cGAS/STING pathway. We also explored the effects of a single-chain variable fragment antibody (scFv T1) and found that scFv T1 alleviated mitochondrial damage and dysfunction by inhibiting the formation of Tau aggregates. These findings suggest that targeting Tau pathology may be crucial to address neuronal mitochondrial impairment and that reduction of the toxicity associated with extracellular Tau aggregates could help slow Tau pathology progression.

## 1. Introduction

Alzheimer’s disease (AD) is the most prevalent neurodegenerative disorder and is characterized primarily by the formation of neurofibrillary tangles resulting from the hyperphosphorylation of Tau proteins [1]. The role of the Tau protein in the pathology of AD has attracted considerable interest. In the neurons of AD patients, Tau proteins dissociate from microtubules and aggregate, which leads to synaptic loss and disrupts axonal transport [2]. Increasing evidence indicates that the Tau aggregates directly contribute to neuronal death and cognitive decline through the mediation of neuroinflammation [3,4]. Clinical studies have shown a strong positive correlation between the severity of Tau pathology and AD progression as well as the deterioration of clinical symptoms [5,6]. This relationship highlights Tau pathology as a crucial target for both the diagnosis and treatment of this disease. Recent discoveries have revealed that extracellular Tau aggregates play a critical role in neurodegenerative diseases such as Alzheimer’s disease initiation and progression. These aggregates not only propagate throughout the brain, exacerbating pathological damage, but they also accelerate neurodegeneration by triggering neuroinflammatory responses and impairing synaptic dysfunction [7,8]. Current research on extracellular Tau aggregates has focused primarily on their mechanisms of pathological transmission, including synaptic connections, exosome-mediated dissemination, and direct cell-to-cell spreading [9,10]. Additionally, the potential of extracellular Tau as an early diagnostic biomarker has garnered significant interest [11]. Nevertheless, the mechanism underlying the toxicity of extracellular Tau aggregates remains unclear.

Mitochondria play essential roles in the nervous system, including maintaining normal energy metabolism, regulating reactive oxygen species (ROS), buffering physiological Ca^2+^ levels, and preserving morphology through mitochondrial dynamics [12]. With advancements in research, mitochondrial dysfunction has emerged as a critical pathophysiological mechanism in AD [13]. Mitochondrial damage directly affects cellular respiration rates, which results in an inadequate energy supply to neurons that cannot meet their energy demands for normal physiological activities, ultimately leading to neuronal death [14,15]. Recent research has demonstrated that mitochondrial dysfunction in AD and related neurodegenerative diseases promotes pathological Tau modifications (including phosphorylation and acetylation), which further exacerbate mitochondrial impairment and establish a self-perpetuating cycle of degeneration [16,17]. Acetylation of Tau protein has been shown to reduce mitochondrial fusion, promote mitochondrial fission, and exacerbate mitochondrial dynamic disorders [18]. Moreover, Tau interacts synergistically with amyloid-beta (Aβ) to expedite neuronal degeneration by triggering the mitochondrial permeability transition pore (mPTP) and facilitating the release of proapoptotic factors, including cytochrome C [19,20]. Quintanilla’s group and Reddy’s group demonstrated that the effect of Tau on mitochondria was caused by the soluble Tau protein produced within the cells [21,22]. Quintanilla’s group has recently shown that caspase-3 cleaved Tau, a pathological modification of Tau, generated mitochondrial dysfunction and cognitive decline by activating mPTP opening in CypD (−/−) knockout mice [23]. Our previous study also demonstrated that extracellular Tau aggregates could act as a pathological seed to enter cells, causing intracellular Tau aggregation and activating cleaved caspase-3 [24]. We agree that the aggregation of intracellular Tau protein is an important mechanism leading to mitochondrial damage. However, extracellular Tau protein, as an important part of the Tau pathological propagation process, requires further investigation into the mechanisms related to mitochondrial damage. Despite the pathological role of Tau, the mechanisms underlying neuronal mitochondrial damage and dysfunction remain poorly understood. Therefore, further investigations into the effects of Tau aggregates, including extracellular Tau aggregates, on the mitochondria of nerve cells are warranted.

Among the intervention strategies that aim to address the complex pathological network of AD, the single-chain variable fragment (scFv) antibody has emerged as a promising treatment modality because of its high specificity, low immunogenicity, and ability to effectively penetrate the blood–brain barrier [25,26,27]. In recent years, scFvs targeting Tau proteins have shown considerable potential in preclinical models for delaying disease progression by inhibiting Tau aggregation, promoting the clearance of pathological Tau, or blocking its transmission between cells [28,29,30]. In our laboratory, an scFv antibody (scFv T1) was screened and found to inhibit the aggregation of Tau in vitro and ameliorate its cytotoxicity in vivo [31].

In this study, we investigated the effects of extracellular Tau aggregates and scFv T1 on the structure and function of mitochondria in SH-SY5Y cells. The results indicate that extracellular Tau aggregates cause mitochondrial damage and dysfunction, which triggers oxidative stress, impairs oxidative phosphorylation, and ultimately leads to neuronal death. ScFv T1 alleviated mitochondrial damage and dysfunction by inhibiting the formation of Tau aggregates. These findings not only clarify the relationship and mechanism between Tau pathology and mitochondrial damage but also provide experimental support and a theoretical foundation for Tau-targeting scFv therapy.

## 2. Materials and Methods

### 2.1. Expression and Purification of Tau Protein

A recombinant plasmid encoding human Tau 40 (residues 1–441) was constructed by subcloning the corresponding DNA fragment into the pET-31b (+) vector. The resulting construct was then transformed into *E. coli* BL21 (DE3) competent cells (TransGen Biotech, Beijing, China, Cat# CD601–02). Recombinant human full-length Tau expression was induced using 0.4 mM IPTG for 16 h at 37 °C. Cells were then lysed by sonication, and the lysate was centrifuged at 12,000 rpm for 20 min at 4 °C. The resulting supernatant was boiled for 10 min and centrifuged again to remove insoluble proteins. After another centrifugation step (12,000 rpm, 20 min, 4 °C), the supernatant was collected and filtered through a 0.45 µm pore-size filter to eliminate insoluble particles. Finally, the soluble protein was purified using a Ni Sepharose 6 Fast Flow column (Cytiva, Marlborough, MA, USA, Cat# 17531801) [24].

### 2.2. Expression and Purification of scFv T1

A recombinant plasmid encoding the scFv T1 was constructed by subcloning the corresponding DNA fragment into a pET-28a (+) vector, which was then transformed into *E. coli* BL21 (DE3). Expression of scFv T1 was induced by adding 1 mM IPTG for 16 h at 16 °C. Cells were then lysed by sonication. The lysate was centrifuged at 12,000 rpm for 20 min at 4 °C, and the resulting supernatant was filtered through a 0.45 µm membrane. The filtered sample was subsequently purified using a Ni Sepharose 6 Fast Flow column (Cytiva Cat# 17531801) [24].

### 2.3. Preparation of Extracellular Tau Aggregates

Tau protein (10 µM) samples were incubated without agitation in aggregation buffer (17.7 mM NaCl, 100 µM DTT, 10 µM heparin and 10 mM HEPES, pH 7.4) at 37 °C for 48 h to obtain extracellular Tau aggregates [32]. Additionally, the Tau protein (10 µM) was incubated with scFv T1 (15 µM) in the same buffer for 48 h at 37 °C to obtain a Tau-scFv T1 mixture [24].

### 2.4. Cell Culture

The SH-SY5Y cells were obtained from Pricella (Pricella Cat# CL-0208, Wuhan, China). SH-SY5Y cells were cultured in DMEM/F12 medium supplemented with 10% fetal bovine serum, 1% penicillin and streptomycin at 37 °C. Furthermore, the cells were supplemented with 5% CO_2_. The cells were divided into several groups and treated with PBS, heparin (5 µM), Tau aggregates (5 µM) or the Tau-scFv T1 mixtures (Tau: 5 µM, scFv T1: 7.5 µM) separately for 24 h [24].

### 2.5. Electron Microscopy of Mitochondrial Ultrastructure

SH-SY5Y cells were treated with PBS, heparin (5 µM), Tau aggregates (5 µM) or the Tau-scFv T1 mixture (Tau: 5 µM, scFv T1: 7.5 µM) for 24 h. The cells were collected and fixed in a fixative solution at 4 °C. The cells were then treated with 1% osmic acid and subsequently encased. Ultrathin sections of the cells were generated and stained with both uranyl acetate and lead citrate. The mitochondrial ultrastructure was observed using a transmission electron microscopy (Ruli TEM, HT7800, Hitachi, Japan). This experiment was performed in triplicate.

### 2.6. Staining of Mitochondria in Living Cells

SH-SY5Y cells were treated with PBS, heparin and different Tau samples for 24 h. The cell culture medium was removed, after which the SH-SY5Y cells were stained for 30 min with staining solution containing 200 nM MitoTracker^®^ probes (Thermo Fisher, Waltham, MA, USA, MitoTracker Green FM, M7514). The mitochondria were observed using laser confocal microscopy (ZEISS LSM700, Oberkochen, Germany).

### 2.7. Western Blot

SH-SY5Y cells were treated with PBS, heparin or different Tau samples for 24 h. The cells were subsequently lysed in RIPA buffer. Total proteins were separated by SDS–PAGE and transferred onto a PVDF membrane (Millipore, Bayswater, Australia, Cat# IPVH00010). These membranes were blocked with QuickBlock™ Blocking Buffer (Beyotime, Shanghai, China, Cat# P0252) followed by overnight incubation with primary antibodies (β-actin antibody, Proteintech, Wuhan, China, Cat No. 66009-1-Ig; Mfn1 antibody, Proteintech, Cat No. 66776-1-Ig; Mfn2 antibody, CST, Danvers, MA, USA, 9482T; Drp1 antibody, Abcam, Cambridge, UK, AB184247; Fis1 antibody, Proteintech, Cat No.66635-1-Ig; Cytochrome C antibody, Abmart, Shanghai, China, T55734F; cGAS antibody, Abmart, PQA3430F; STING antibody, Abmart, TD12090F; VDAC1, CST, 4661T; GAPDH, proteintech, Cat No. 10494-1-AP; Histone H3, proteintech, Cat No. 17168-1-AP). The membranes were subsequently washed three times with TBST for 10 min each time. The membranes were then incubated with HRP-conjugated secondary antibodies (goat anti-mouse HRP-conjugated, ABclonal, Wuhan, China, AS003; goat anti-rabbit HRP-conjugated, Proteintech Cat# SA00001-2) for 1 h at RT and washed 3 times with TBST for 15 min each time. Finally, the bands were visualized with a chemiluminescent HRP substrate (Millipore, Cat# P90719) and analyzed using a ChemiDocTM XRS+ system with Image LabTM Software version 3.0 (Bio-Rad, Hercules, CA, USA, ChemiDoc MP) [24]. This experiment was performed in triplicate. To calculate the fold change in protein expression, the ratio of target protein to β-actin in each group was first determined, and then normalized to the value obtained from the NC group.

### 2.8. ROS Assay

SH-SY5Y cells were divided into several groups and treated with PBS, heparin, or different Tau samples for 24 h. ROS levels were detected with an ROS detection kit (Beyotime Cat# S0033S). The cells were first incubated with the probe CM-H_2_DCFH-DA for 20 min at 37 °C after which they were then collected and examined by flow cytometry. The experiment was performed in triplicate, and each experiment included 10,000 cells per replicate. The statistical analyses were performed with GraphPad Prism 8 software.

### 2.9. Apoptosis Assay

SH-SY5Y cells were divided into several groups and treated with PBS, heparin or different Tau samples for 24 h. The cells were collected and stained with propidium iodide (PI) and Annexin V-FITC (Beyotime Biotechnology Co., Ltd.), after which the cells were filtered and examined by flow cytometry (ACEA NovoCyte3130, ACEA Biosciences, San Diego, CA, USA) using an ACEA flow cytometry analysis system (Novo Express version 1.6.2). This experiment was repeated three times, and each experiment performed included 10,000 cells per replicate. The statistical analyses were performed with GraphPad Prism 8 software.

### 2.10. Biochemical Index Assay

SH-SY5Y cells were divided into several groups and treated with PBS, heparin or different Tau samples for 24 h. Biochemical index assays were performed using kits for glutathione (GSH) (Beyotime, S0052), total antioxidant capacity (T-AOC) (Beyotime, S0121) and superoxide dismutase (SOD) (Beyotime, S0101S) according to the manufacturer’s instructions. This experiment was repeated three times, and each sample was analyzed in triplicate.

### 2.11. qPCR Assay

SH-SY5Y cells were divided into several groups and treated with PBS, heparin or different Tau samples for 24 h. The total DNA of the SH-SY5Y cells was purified using a DNA extraction kit. In addition, the mitochondria were separated with a cell mitochondria isolation kit (Beyotime, C3601), and the isolated cytoplasmic DNA was purified with a DNA isolation kit. Real-time qPCR was performed using PerfectStart Green qPCR SuperMix (TransGen Biotech Cat# AQ602) and an ABI Quant Studio 6 Flex Real-Time PCR System (Thermo Fisher, QuantStudio™ 6). Total RNA was extracted from each group of cells and subsequently reverse-transcribed into cDNA for qPCR analysis. The expression levels of the respective genes were analyzed in triplicate for each sample. qPCR was used to detect the levels of expression of mtDNA [33], *CXCL10*, *IL-6*, *Bax* and *Bcl-2* (Table 1).

### 2.12. Mitochondrial Membrane Potential (MMP) Assay

SH-SY5Y cells were divided into several groups and treated with PBS, heparin or different Tau samples for 24 h. The cells were then subjected to JC-1 staining (Beyotime, C1071S) for 1 h at 37 °C in the dark and observed by laser confocal microscopy (ZEISS LSM700). In addition, the cells in each group were subjected to TMRM staining (MedChemExpress, Shanghai, China, HY-D0984A) for 30 min at 37 °C in the dark [34]. Then the cells were stained using Hoechst 33342 living cell nucleus staining solution (Beyotime, C1027), and observed by laser confocal microscopy (ZEISS LSM700). The experiment was performed in triplicate. Fluorescence intensity quantitative analysis of the mitochondrial membrane potential in SH-SY5Y cells was performed via ImageJ software version 2.9.0.

### 2.13. Seahorse XF Cell Mito Stress Test

SH-SY5Y cells were divided into several groups and treated with PBS, heparin or different Tau samples for 24 h. The mitochondrial functions of the cells were measured via a Seahorse XF Cell Mito Stress Test Kit (103015-100, Agilent Technologies, Santa Clar, CA, USA) and a Seahorse XF24 extracellular flux analyzer. Mitochondrial complex inhibitors (oligomycin (1.5 µM), FCCP (1 µM) and rotenone/antimycin A (1 µM)) were successively added to the cell culture microplate to measure the oxygen consumption rate and key parameters of mitochondrial function using a Seahorse XF24 analyzer version 2.6.4. Each sample was assayed with a minimum of five replicates, and the data were normalized to the number of cells in each well.

### 2.14. ATP Assay

SH-SY5Y cells were divided into several groups and treated with PBS, heparin or different Tau samples for 24 h. ATP levels in the cells were measured with an ATP assay kit (Beyotime, S0026). Each sample was assayed with a minimum of three replicates.

### 2.15. Immunofluorescence

Cells grown on coverslips were fixed with 4% paraformaldehyde for 20 min at room temperature, permeabilized with 0.2% Triton X-100 for 10 min, and blocked with 5% normal donkey serum for 1 h. Samples were then incubated overnight at 4 °C with rabbit anti-TFAM (1:200, ABclonal, A23467) and mouse anti-TOM20 (1:200, ABclonal, A27799). After washing with PBS, cells were incubated with a mixture of Alexa Fluor 488-conjugated goat anti-mouse (1:500, Abcam, ab150113) and Alexa Fluor 594-conjugated donkey anti-rabbit (1:500, Jackson, Susquehanna, PA, USA, 711-585-152) secondary antibodies for 1 h at room temperature in the dark. Nuclei were counterstained with DAPI for 5 min. Coverslips were mounted onto slides using an anti-fade mounting medium. Images were acquired using a confocal microscope (ZEISS LSM880) and processed with ZEN software version 2.3 (ZEISS) and ImageJ.

### 2.16. Statistical Analysis

All the statistical analyses were performed with GraphPad Prism 8.0 software. Data are presented as mean ± SEM. Statistical analysis was performed using one-way ANOVA followed by Tukey’s multiple comparisons test to assess differences between groups, and Student’s *t* test was used to analyze the differences between the two groups. A *p*-value < 0.05 was considered significant. * *p* < 0.05, ** *p* < 0.01, and *** *p* < 0.001.

## 3. Results

### 3.1. Extracellular Tau Aggregates Cause Mitochondrial Damage and Dynamics Disorder

**Figure 1:**
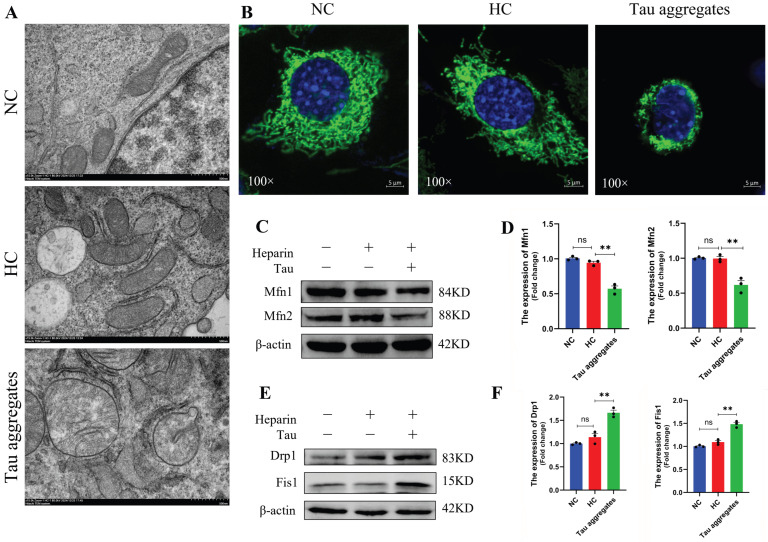
Effects of extracellular Tau aggregates on mitochondrial damage and mitochondrial dynamic disorders in SH-SY5Y cells. (**A**) Representative electron micrograph of mitochondrial morphology. The image was taken at a magnification of 15,000× (the scale bar corresponds to 500 nm). (**B**) Representative confocal micrograph of mitochondria. Scale bar, 5 μm. (**C**) Expression levels of Mfn1 and Mfn2 in SH-SY5Y cells detected by Western blot. The grouping of blots cropped from different gels of the same samples. (**D**) Bar chart of the relative protein expression levels in (**C**) (n = 3). (**E**) Expression levels of Drp1 and Fis1 in SH-SY5Y cells detected by Western blot. The grouping of blots cropped from different gels of the same samples. (**F**) Bar chart of the relative protein expression levels in (**E**) (n = 3).

Mitochondrial damage is an important event in AD. As mitochondria are important energy metabolism organelles within cells, abnormalities in mitochondria directly lead to neuronal damage [35]. The full-length human Tau protein was purified (Figure S1) and used in our study. To investigate the effects of extracellular Tau aggregates on mitochondria, aggregation of Tau monomers was induced by heparin in vitro, and SH-SY5Y cells were then treated with PBS, heparin or induced Tau aggregates. The structure of the mitochondria was detected by transmission electron microscopy. The results showed that the mitochondria in the PBS-treated normal control (NC) group and the heparin-treated control (HC) group contained well-preserved surrounding membranes and well-defined cristae (Figure 1A). However, mitochondrial swelling and vacuolation were observed in the Tau aggregates treatment group (Figure 1A). The mitochondria of cells in the NC group and the HC group were tubular. The mitochondria of cells in the Tau aggregates treatment group were aggregated (Figure 1B). We next investigated whether treatment with extracellular Tau aggregates disrupted the mitochondrial dynamics of SH-SY5Y cells. Mitochondrial dynamics-related proteins were detected by Western blot, and the results showed that the expression levels of Mfn1 and Mfn2 were lower in the Tau aggregates treatment group than in the NC and HC groups (Figure 1C,D), whereas the expression levels of Drp1 and Fis1 were greater in the Tau aggregates treatment group than in the NC and HC groups (Figure 1E,F). These results indicate that extracellular Tau aggregates cause mitochondrial damage and dynamics disorders in SH-SY5Y cells.

### 3.2. Extracellular Tau Aggregates Cause Oxidative Stress

**Figure 2:**
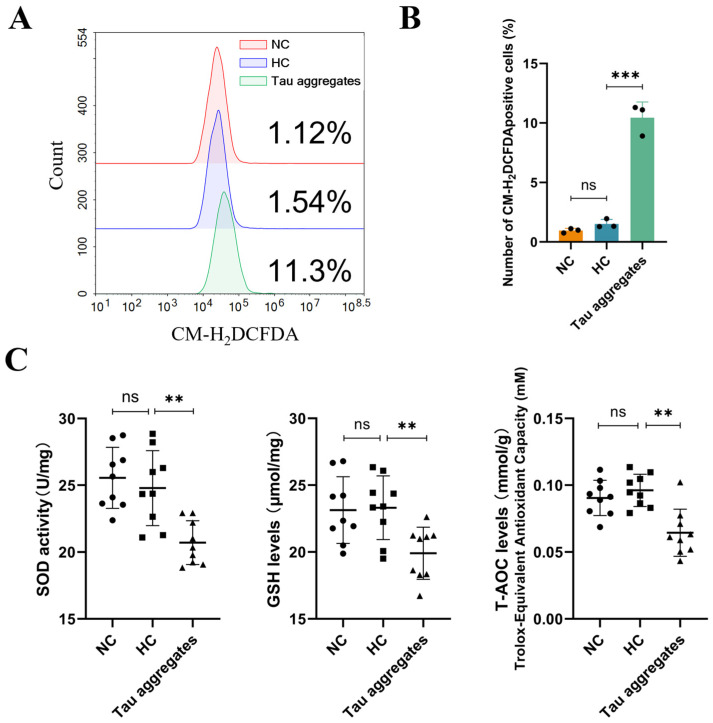
Effects of extracellular Tau aggregates on mitochondrial oxidative stress in SH-SY5Y cells. (**A**) ROS levels in cells measured by flow cytometry. (**B**) Number of CM-H_2_DCFDA-positive cells (n = 3). (**C**) SOD, GSH and T-AOC levels were detected using biochemical index assays (n = 9).

Mitochondrial damage usually leads to a decrease in the mitochondrial membrane potential (MMP) and an increase in ROS production, which further causes oxidative stress [36]. In our study, flow cytometry was performed to examine the ROS levels in SH-SY5Y cells. The results showed that the ROS levels in the Tau aggregates treatment group were significantly greater than those in the NC and HC groups (Figure 2A,B). SOD, GSH and T-AOC are factors related to oxidative stress, and thus the levels of these factors were detected using biochemical index assays. The results showed no significant difference in the levels of SOD, GSH and T-AOC between cells in the NC and HC groups, but the levels of these factors were significantly decreased in the Tau aggregates treatment group (Figure 2C). These results indicate that extracellular Tau aggregates cause mitochondrial oxidative stress.

### 3.3. Extracellular Tau Aggregates Disrupt Oxidative Phosphorylation and Inhibit Energy Production in Mitochondria

**Figure 3:**
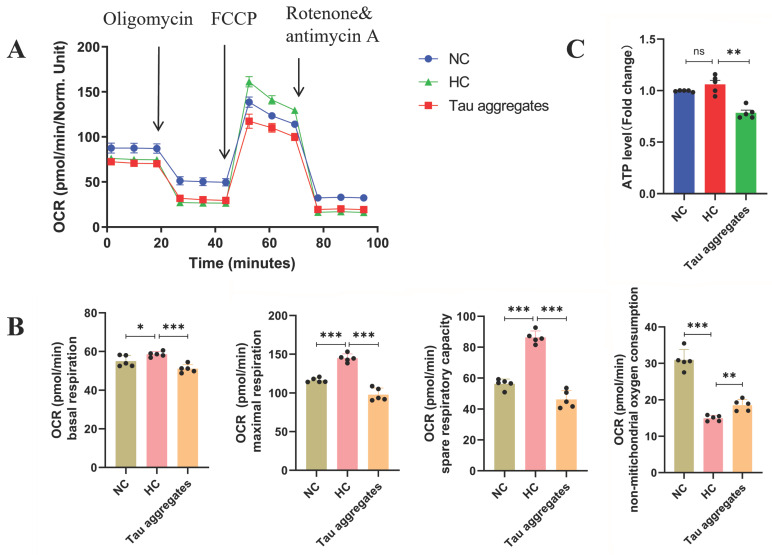
Effects of extracellular Tau aggregates on mitochondrial oxidative phosphorylation and ATP production. (**A**) Seahorse tracing of the oxygen consumption rate (OCR) in SH-SY5Y cells. (**B**) Bar chart of the spare respiratory capacity, maximal respiration, basal respiration and non-mitochondrial oxygen consumption (n = 5). (**C**) ATP levels in SH-SY5Y cells (n = 5).

Mitochondrial oxidative phosphorylation is crucial for providing energy to neurons. Disorders of mitochondrial energy metabolism induced by mitochondrial damage and oxidative stress are related to cell death [37]. The oxygen consumption rate (OCR) directly reflects the level of mitochondrial oxidative phosphorylation in cells. In this study, the OCR was detected via the Seahorse XF Cell Mito Stress Test. As shown in Figure 3A,B, the basal respiration, maximal respiration and spare respiratory capacity of the cells in the HC group were greater than those in the NC group. Most importantly, basal respiration, maximal respiration and spare respiratory capacity were significantly lower in the Tau aggregates treatment group than in the HC group. In contrast, non-mitochondrial oxygen consumption in the Tau aggregates treatment group was greater than that in the HC group. The ATP level in the Tau aggregates treatment group was significantly lower than that in the HC group (Figure 3C). These results indicate that the mitochondrial oxidative phosphorylation in SH-SY5Y cells is disrupted and ATP production is inhibited after treatment with extracellular Tau aggregates.

### 3.4. Mitochondrial Damage and Dysfunction Induced by Extracellular Tau Aggregates Are Important Factors in SH-SY5Y Cell Death

**Figure 4:**
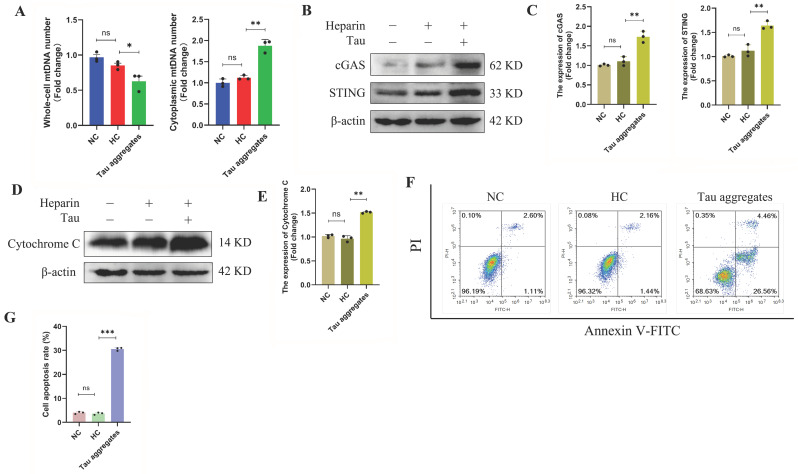
Extracellular Tau aggregates cause SH-SY5Y cells to undergo apoptosis. (**A**) mtDNA levels in whole cells or the cytoplasm were detected by real-time qPCR (n = 3). (**B**) Expression of the cGAS and STING proteins was detected by Western blot. The grouping of blots cropped from different gels of the same samples. (**C**) Relative expression levels of cGAS and STING in each group (n = 3). (**D**) Expression of cytoplasmic cytochrome C detected by Western blot. The grouping of blots cropped from different gels of the same samples. (**E**) Relative expression level of cytochrome C in each group (n = 3). (**F**) Apoptosis of SH-SY5Y cells detected by flow cytometry. (**G**) Apoptosis rates of SH-SY5Y cells in each group (n = 3).

Many mechanisms, including oxidative stress and mitochondrial dysfunction, regulate cell death. When mitochondria are destroyed, mitochondrial DNA (mtDNA) leaks into the cytoplasm, which activates the cGAS/STING signaling pathway and induces cell death [38,39]. In this study, whole cell lysate (total), mitochondrial fraction (mito), and cytosolic fraction (cyto) were analyzed by Western blotting using antibodies against VDAC (mitochondrial marker), GAPDH (cytosolic marker), and Histone H3 (nuclear marker). VDAC was exclusively detected in the total cell lysate and mitochondrial fractions but absent from the cytosolic fraction. GAPDH is predominantly localized in the total cell lysate and cytoplasmic fraction, with only trace amounts detected in the mitochondrial fraction. Histone H3 was only detected in the total cell lysate (Figure S2). MtDNA leakage and the expression levels of proteins in the cGAS/STING signaling pathway were detected. The results showed that the whole-cell mtDNA levels in the SH-SY5Y cells in the Tau aggregates treatment group were lower than those in the cells in the HC group. However, the cytoplasmic mtDNA levels in the cells in the Tau aggregates treatment group were significantly greater than those in the cells in the HC group (Figure 4A). The results of the immunofluorescence showed that the mtDNA in the cells of the Tau aggregates treatment group was significantly increased compared with the HC group (Figure S3). In addition, the protein expression levels of cGAS and STING in the cells in the Tau aggregates treatment group were significantly greater than those in the HC group (Figure 4B,C). *CXCL10* and *IL-6* are downstream target genes of the cGAS/STING signaling pathway. *Bax* and *Bcl-2* are also indirectly affected by cGAS/STING. Therefore, we used qPCR to detect the expression levels of *CXCL10*, *IL-6*, *Bax* and *Bcl-2*. The mRNA levels of *CXCL10*, *IL-6* and *Bax* in the Tau aggregates treatment group were significantly higher than those in the HC group, whereas *Bcl-2* mRNA levels were significantly lower (Figure S4). In response to cellular damage, such as oxidative stress or DNA breakage, cytochrome C is released from the inner mitochondrial membrane into the cytosol, where it initiates the caspase cascade, thereby inducing apoptosis [40]. The level of cytoplasmic cytochrome C was detected by Western blot. The results showed that the cytochrome C level in the Tau aggregates treatment group was greater than that in the HC group (Figure 4D,E). By flow cytometry, we found that the apoptosis rates of SH-SY5Y cells treated with Tau aggregates were significantly greater than those of the cells in the NC and HC groups (Figure 4F,G). These results indicate that once extracellular Tau aggregates cause mitochondrial damage, mtDNA is released into the cytoplasm where it activates the cGAS/STING signaling pathway and the level of cytochrome C is increased, which eventually leads to cell death.

### 3.5. ScFv T1 Alleviates Cell Death by Reducing Extracellular Tau Aggregate-Induced Mitochondrial Damage and Dysfunction

**Figure 5:**
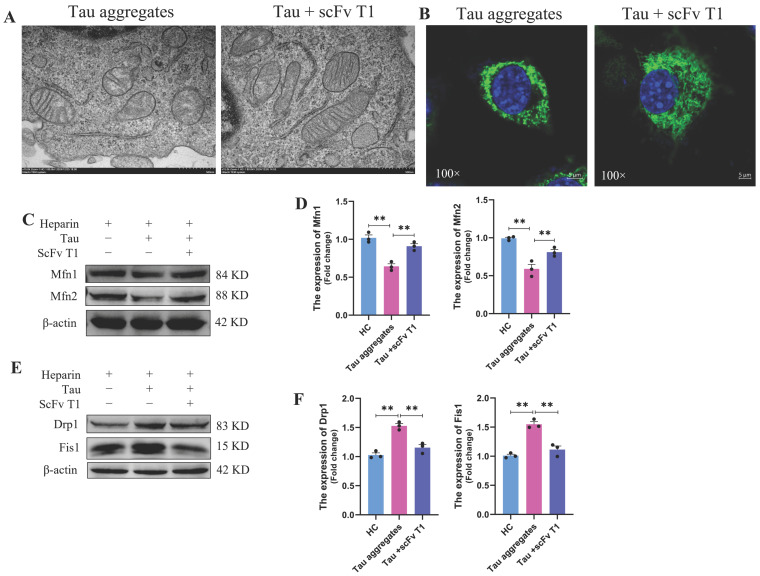
Effects of scFv T1 on SH-SY5Y cell mitochondrial damage and mitochondrial dynamic disorders caused by extracellular Tau aggregates. (**A**) Representative electron micrograph of mitochondrial morphology in SH-SY5Y cells. The image was taken at a magnification of 15,000× (the scale bar corresponds to 500 nm). (**B**) Representative confocal micrograph of mitochondria in SH-SY5Y cells. Scale bar, 5 μm. (**C**) Expression levels of Mfn1 and Mfn2 in SH-SY5Y cells, as detected by Western blot. The grouping of blots cropped from different gels of the same samples. (**D**) Bar chart of the relative protein expression levels in (**C**) (n = 3). (**E**) Expression levels of Drp1 and Fis1 in SH-SY5Y cells, as detected by Western blot. The grouping of blots cropped from different gels of the same samples. (**F**) Bar chart of the relative protein expression levels in (**E**) (n = 3).

In previous studies, we reported that scFv T1 inhibits the aggregation of Tau and reduces its neurocytotoxicity [24,31]. In this study, scFv T1 was also expressed and purified (Figure S5). To explore the protective effect of scFv T1 on Tau aggregate-induced mitochondrial damage and dysfunction, the Tau monomers were incubated with scFv T1 for 48 h, and the SH-SY5Y cells were treated with the Tau-scFv T1 mixtures. As shown in Figure 5A,B, mitochondrial swelling, vacuolation and the degree of mitochondrial aggregation were alleviated in the Tau-scFv T1 mixtures treatment group. We next measured the expression levels of mitochondrial dynamics-related proteins. The results showed that the expression levels of Mfn1 and Mfn2 in the Tau-scFv T1 mixtures treatment group were significantly greater than those in the Tau aggregates treatment group, whereas the expression levels of Drp1 and Fis1 were lower in the Tau-scFv T1 mixtures treatment group than in the Tau aggregates treatment group (Figure 5C–F). These findings indicate that mitochondrial dynamics disorder were alleviated after inhibition of Tau aggregate formation by scFv T1.

**Figure 6:**
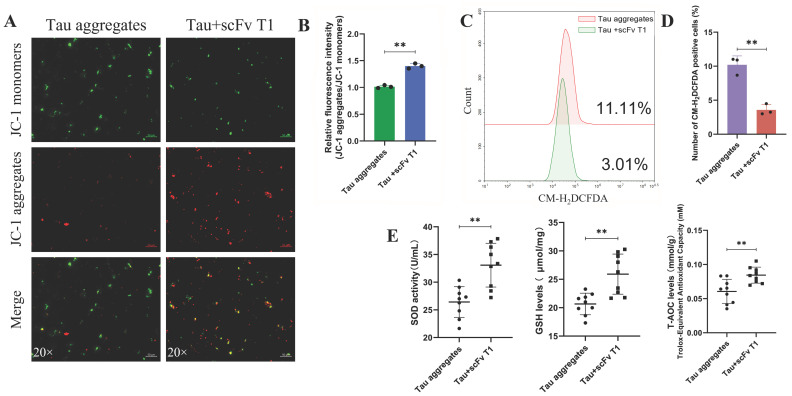
Effects of scFv T1 on mitochondrial oxidative stress induced by extracellular Tau aggregates. (**A**) Mitochondrial membrane potential indicated by JC-1-specific fluorescence. Scale bar, 50 μm. (**B**) Red/green fluorescence intensity ratios of the cells in each group (n = 3). (**C**) ROS levels in cells measured by flow cytometry. (**D**) Number of CM-H_2_DCFDA-positive cells (n = 3). (**E**) SOD, GSH and T-AOC levels were detected using biochemical index assays (n = 9).

**Figure 7:**
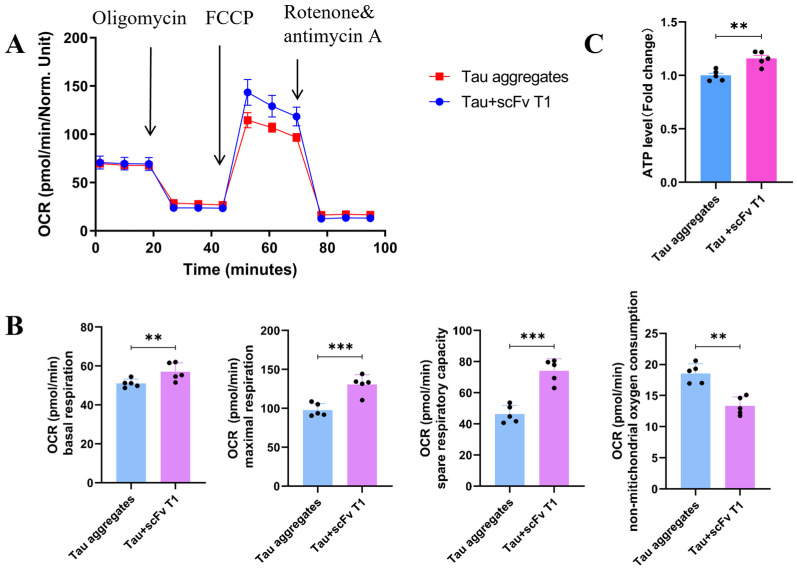
Effects of scFvT1 on mitochondrial oxidative phosphorylation dysfunction induced by extracellular Tau aggregates. (**A**) Seahorse tracing of the oxygen consumption rate (OCR) in SH-SY5Y cells. (**B**) Bar chart of the spare respiratory capacity, maximal respiration, basal respiration and non-mitochondrial oxygen consumption (n = 5). (**C**) ATP levels in SH-SY5Y cells (n = 5).

The effects of scFv T1 on Tau aggregate-induced oxidative stress and cell death were also determined in our study. The results showed that the MMP of the SH-SY5Y cells in the Tau-scFv T1 mixtures treatment group was greater than that of the cells in the Tau aggregates treatment group (Figure 6A,B and Figure S6). Additionally, the ROS level of the cells was significantly decreased in the Tau-scFv T1 mixtures treatment group (Figure 6C,D), whereas the levels of SOD, GSH and T-AOC were increased (Figure 6E). The basal respiration, maximal respiration, spare respiratory capacity and ATP levels of SH-SY5Y cells were increased in the Tau-scFv T1 mixtures treatment group (Figure 7A–C), whereas the non-mitochondrial oxygen consumption was decreased (Figure 7A,B).

**Figure 8:**
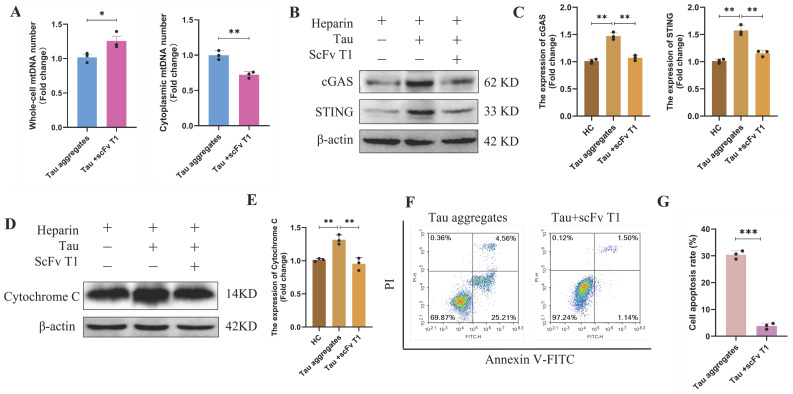
Effects of scFv T1 on the apoptosis of SH-SY5Y cells induced by extracellular Tau aggregates. (**A**) mtDNA levels in whole cells or the cytoplasm, as detected by real-time qPCR (n = 3). (**B**) Expression of the cGAS and STING proteins was detected by Western blot. The grouping of blots cropped from different gels of the same samples. (**C**) Relative expression levels of cGAS and STING in each group (n = 3). (**D**) Expression of cytoplasmic cytochrome C detected by Western blot. The grouping of blots cropped from different gels of the same samples. (**E**) Relative expression level of cytochrome C in each group (n = 3). (**F**) Apoptosis of SH-SY5Y cells, as detected by flow cytometry. (**G**) Apoptosis rates of SH-SY5Y cells in each group (n = 3).

The cytoplasmic mtDNA and cytochrome C of the cells in the Tau-scFv T1 mixtures treatment group were decreased, and activation of the cGAS/STING signaling pathway was inhibited (Figure 8A–E and Figure S3); the mRNA levels of *CXCL10*, *IL-6*, and *Bax* were significantly decreased, while the mRNA level of *Bcl-2* was significantly increased (Figure S4). The flow cytometry results showed that the apoptosis rates of the SH-SY5Y cells in the Tau-scFv T1 mixture treatment group were significantly lower than those of the cells in the Tau aggregates treatment group (Figure 8F,G). These results indicate that scFv T1 alleviates neuronal death by reducing extracellular Tau aggregate-induced mitochondrial damage and dysfunction and reducing mitochondrial oxidative stress.

## 4. Discussion

AD is a progressive neurodegenerative disorder characterized by the pathological accumulation of Tau protein within neurons. The neurodegenerative cascade is partially initiated through aberrant phosphorylation of the microtubule-associated protein Tau, which promotes its dissociation from microtubules and subsequent self-assembly into β-sheet-rich fibrillar aggregates [41]. Intracellular Tau aggregates disrupt cellular proteostasis by impairing proteasome function such as through sequestering molecular chaperones including HSP70 [42]. Additionally, these aggregates compromise synaptic plasticity by interfering with the localization and function of postsynaptic receptors [43]. Notably, the stereotypical progression of AD pathology across brain regions exhibits a striking spatiotemporal correlation with prion-like transneuronal transmission of pathological Tau species [44]. During disease progression, degenerating neurons release propagative Tau seeds through exosomal trafficking and membrane rupture, which enables activity-dependent endocytic uptake by connected neurons [45,46]. The emerging consensus suggests that low-molecular-weight oligomeric species are the primary effectors of synaptotoxicity [47,48]. The neurotoxic potential of extracellular Tau aggregates has emerged as a significant focus in neurodegenerative disease research. On the one hand, extracellular Tau aggregates spread between neurons in a “prion-like” manner, inducing abnormal aggregation of normal Tau proteins [49]. On the other hand, they activate glial cells to establish inflammation and thereby cause neuronal damage [50]. Despite the well-established correlation between extracellular Tau aggregates and disease progression, the precise mechanisms underlying Tau-mediated neurodegeneration remain elusive. To address this knowledge gap, we employed heparin-induced Tau aggregates as an experimentally validated model system [51] to investigate the pathogenic mechanism of extracellular Tau aggregates in AD.

Mitochondrial function is fundamental to neuronal physiology, and mounting evidence has highlighted the intimate connection between mitochondrial dysfunction and AD pathogenesis [52,53]. Mitochondrial integrity is the basis of mitochondrial metabolic activity and health. In addition, mitochondria are highly dynamic organelles that undergo coordinated cycles of fission and fusion, which are essential for maintaining mitochondrial homeostasis [54,55]. In this study, we discovered that extracellular tau aggregates destroy the structure of mitochondrial cristae and accelerate mitochondrial vacuolation. Cristae morphology was assessed qualitatively using representative high-magnification TEM images from multiple independent fields, and future studies using electron tomography or FIB-SEM will be required for definitive quantification. Moreover, extracellular Tau aggregates impair the balance between mitochondrial fission and fusion. These adverse effects compromise mitochondrial integrity and may exacerbate mitochondrial dysfunction, such as loss of the mitochondrial membrane potential, increased ROS generation, respiratory chain dysfunction and reduced ATP production.

MMP serves as a crucial indicator of mitochondrial health. Elevated ROS levels significantly diminish the MMP, although it can recover upon the removal of ROS [56,57]. Excessive production of ROS, as byproducts of mitochondrial activity, can lead to oxidative stress [58], which ultimately results in neuronal damage. Notably, mitochondria possess an intrinsic antioxidant defense system comprising GSH and enzymes such as SOD, which neutralize excessive ROS and maintain cellular redox homeostasis [59]. Sun et al. reported that Tau oligomers caused a decrease in MMP [60]. Cente et al. demonstrated that the presence of truncated Tau proteins leads to the accumulation of ROS and increases the sensitivity of neurons to stress-induced cell death in a Tau disease model in transgenic rats [61]. In previous studies, we found that Tau aggregates caused the accumulation of ROS using DCFH-DA staining, and ROS further promote the intracellular aggregation of Tau protein [24]. In this study, we confirmed this finding by flow cytometry analysis. We also found some antioxidant activities were decreased after 24 h of treatment with extracellular Tau aggregates. These results indicate that extracellular Tau aggregates can damage mitochondria and cause oxidative stress in cells.

The efficient operation of the nervous system requires high energy. Mitochondria serve as the primary energy source for neurons, as they convert nutrients into ATP through oxidative phosphorylation. Increasing numbers of studies have shown that energy metabolism in neurons is abnormal in AD [62]. Our study revealed that heparin can partially enhance mitochondrial respiration; however, its use as a negative control does not affect the exploration of the effects of extracellular Tau aggregates on mitochondrial respiration. The results of our study revealed that extracellular Tau aggregates disrupt the oxidative phosphorylation process in neuronal mitochondria and inhibit mitochondrial respiration and ATP production, thereby forcing cells to resort to non-mitochondrial respiration for their energy supply.

Multiple mitochondria-related mechanisms within cells, including mtDNA leakage and increased level of cytochrome C, regulate cell death. Cell death is accompanied by activation of the cGAS/STING signaling pathway, which is usually activated by mtDNA leakage [63]. Furthermore, some studies have shown that the increase in cytochrome C levels or its ubiquitylation promotes neuronal apoptosis [64,65]. In this study, we observed that after mitochondria were damaged by extracellular Tau aggregates, mtDNA was released into the cytoplasm, which activated the cGAS/STING signaling pathway. Activation of the cGAS/STING pathway leads to the activation of downstream *IL-6*, while the pro-apoptotic factor *Bax* and the anti-apoptotic *Bcl-2* are affected. Moreover, the intracellular cytochrome C levels increased. As a result, the apoptosis rates of SH-SY5Y cells significantly increased after treatment with extracellular Tau aggregates.

Currently, the treatment of AD remains a significant global challenge, and antibody-based therapies targeting Tau pathology have emerged as promising therapeutic strategies [66,67]. Most of the current anti-Tau antibodies are designed to target phosphorylated epitopes or oligomeric species and function by inhibiting the intercellular propagation of Tau and by enhancing Tau clearance [68,69,70]. We previously screened a single-chain variable fragment antibody (scFv T1) that could inhibit the aggregation of Tau in vitro and ameliorate its cytotoxicity. Our findings in the current study demonstrated that scFv T1 significantly mitigated the detrimental effects of extracellular Tau aggregates on SH-SY5Y cells’ mitochondria, alleviated the suppression of mitochondrial respiration, reduced oxidative stress and decreased neuronal death. These results highlight the therapeutic potential of scFv T1 in ameliorating Tau-induced mitochondrial dysfunction and mitochondria-related neurodegeneration.

In conclusion, we systematically investigated the impact of extracellular Tau aggregates on the structural integrity and functional capacity of neuronal mitochondria. These results indicate that extracellular Tau aggregates not only lead to the destruction of mitochondrial structure and mitochondrial dynamic disorders but also cause mitochondrial oxidative stress and the inhibition of oxidative phosphorylation in neurons. These Tau samples promote neuronal death through a reduction in ATP production, the increase in cytochrome C, mtDNA leakage and activation of the cGAS/STING pathway. Furthermore, we found that scFv T1 can effectively alleviate mitochondrial damage and dysfunction by inhibiting Tau aggregation. These findings advance our understanding of the relationship between mitochondrial damage and Tau-related diseases pathogenesis and provide a novel therapeutic direction for antibody-based interventions targeting Tau pathology. Several limitations should be acknowledged, particularly regarding the experimental model used. Although SH-SY5Y cells are widely employed in neurological disease research, they are not fully representative of primary neurons. To address this limitation and enhance the translational relevance of our findings, future studies will incorporate complementary models, such as primary neuronal cultures or in vivo mouse systems. We did not directly intervene in the cGAS/STING signaling pathway to confirm that this pathway is an independent event. By blocking the cGAS/STING pathway through genetic or pharmacological means, it will be possible to determine whether this pathway can be regarded as a potential therapeutic target and be utilized for intervention treatment. In addition, we adopted a preventive approach to investigate the function of scFv T1, which is also a limitation of this study. In the future, we will adopt a therapeutic approach to investigate the reversing effect of scFv T1 on the damage caused by Tau aggregates.

## Figures and Tables

**Table 1:** All gene primer information for qPCR.

Primers	Primers Sequences	Product Length (bp)	Annealing Temp. (°C)	Gene Accession Number
mtDNA [33]	CAAACCTACGCCAAAATCCA	164	54	NC_012920.1
	GAAATGAATGAGCCTACAGA			
*β-actin*	TTAATAGTCATTCCAAATATGA	246	54	NC_000007.14
	GGGACAAAAAAGGGGGAAGG			
*CXCL10*	GCTTCCAAGGATGGACCACA	253	60	NM_001565.4
	GCAGGGTCAGAACATCCACT			
*IL-6*	GTCCAGTTGCCTTCTCCCTGG	617	60	NM_000600.5
	CCCATGCTACATTTGCCGAAG			
*Bax*	TGATGGACGGGTCCGGG	422	60	NM_001291428.2
	TGTCCAGCCCATGATGGTTC			
*Bcl-2*	AAAAATACAACATCACAGAGGAAGT	738	60	NM_000633.3
	CAATCCTCCCCCAGTTCACC			

## Data Availability

The original contributions presented in this study are included in the article/Supplementary Materials. Further inquiries can be directed to the corresponding authors.

## Supplementary Materials

The following supporting information can be downloaded at https://www.mdpi.com/article/10.3390/antiox15040515/s1: Figure S1: SDS-PAGE analysis of Tau protein purification; Figure S2: Validation of subcellular fractionation purity; Figure S3: TFAM and TOM20 immunofluorescence showing mtDNA release; Figure S4: qPCR analysis of cGAS-STING downstream genes; Figure S5: SDS-PAGE analysis of scFv T1 purification; Figure S6: Mitochondrial membrane potential indicated by TMRM fluorescence.
