# Supplementary material for: ScFv T1 Protects Against Mitochondrial Damage of SH-SY5Y Cells Caused by Extracellular Tau Aggregates
Source: Antioxidants (Basel). 2026 Apr 21;15(4):515. doi: 10.3390/antiox15040515 (PMC13113621; doi:10.3390/antiox15040515)
Supplement: Supplementary file 1 [file antioxidants-15-00515-s001.zip › antioxidants-4184920-supplementary.pdf]

Supplemental Material for "**ScFv T1 protects against mitochondrial damage of SH-SY5Y cells caused by extracellular Tau aggregates**"

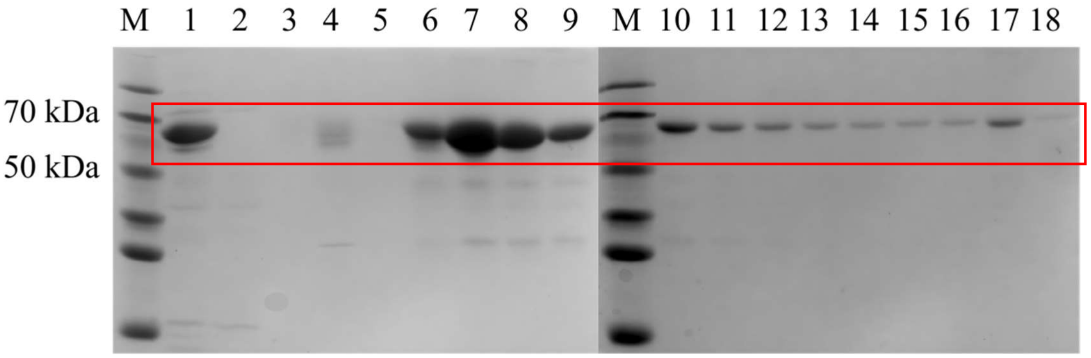

Figure S1. SDS-PAGE analysis of Tau protein purification. M: Marker; 1: The sample after the expression bacteria have been lysed; 2: Flow-through liquid sample; 3: Balanced solution sample; 4-5: Buffer solution containing 40 mM imidazole; 6-10: Elution solution containing 100 mM imidazole; 11-16: Elution solution containing 200 mM imidazole; 17-18: Elution solution containing 500 mM imidazole

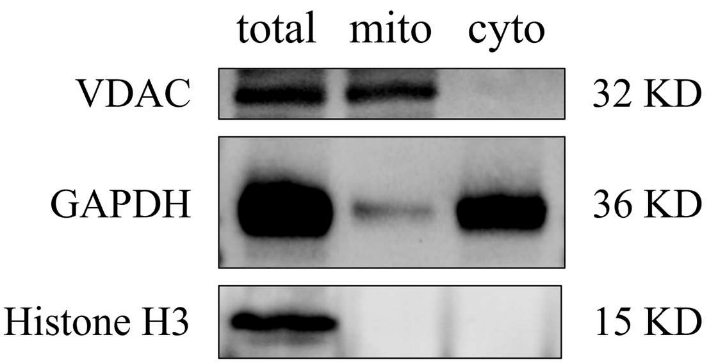

Figure S2. Validation of subcellular fractionation purity. Western blot analysis of whole cell lysate (total), mitochondrial (mito), and cytosolic (cyto) fractions using antibodies against VDAC (mitochondrial marker), GAPDH (cytosolic marker), and Histone H3 (nuclear marker)

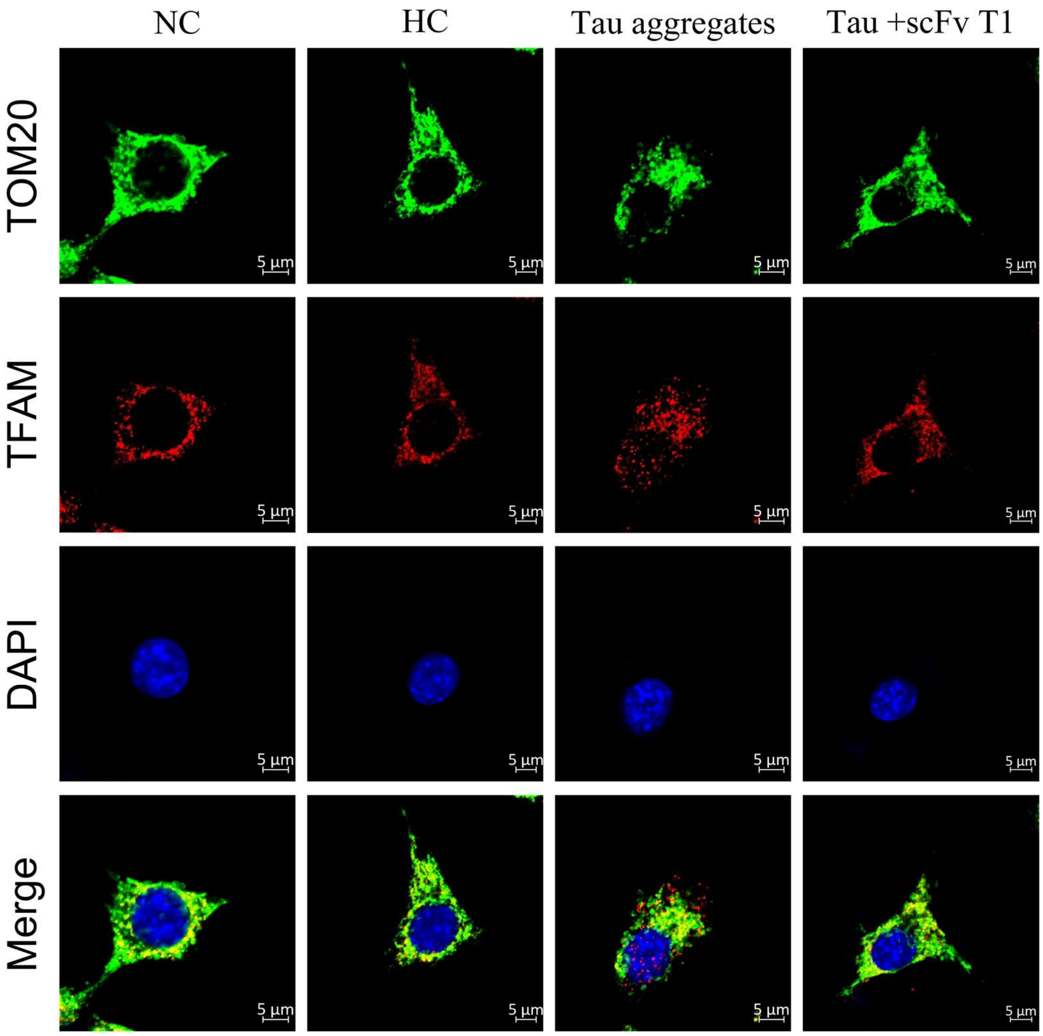

Figure S3. TFAM and TOM20 immunofluorescence showing mtDNA release. Representative IF images of TOM20 (green, mitochondria), TFAM (red, mtDNA), and DAPI (blue, nuclei). Scale bar, 5 μm

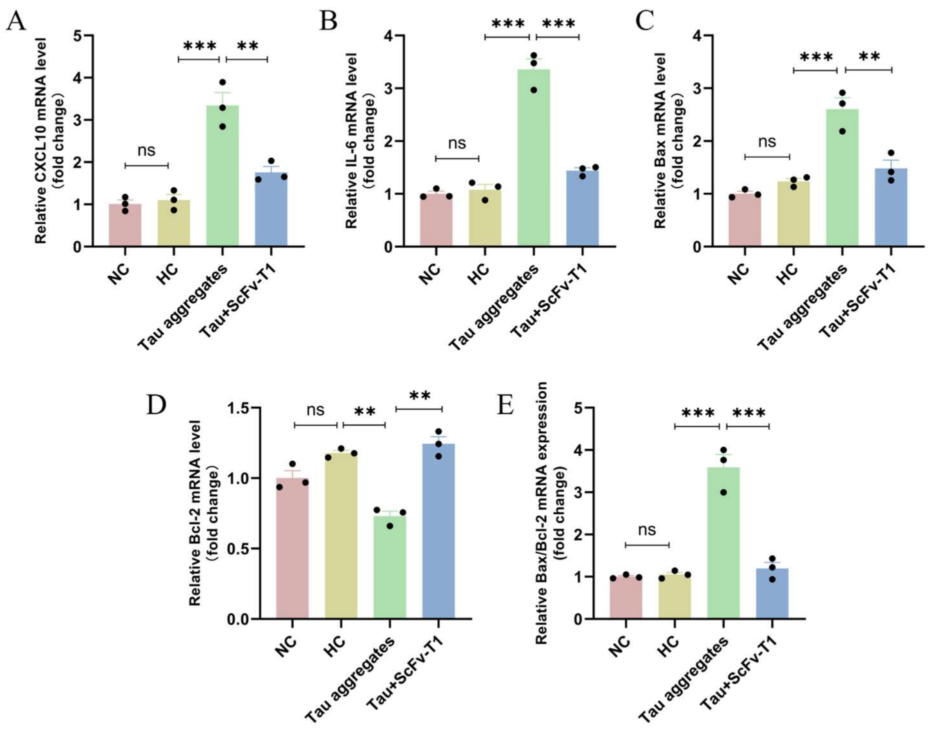

Figure S4. qPCR analysis of cGAS-STING downstream genes. The mRNA expression levels of CXCL10 (A), IL-6 (B), Bax (C), and Bcl-2 (D) in SH-SY5Y cells were measured by qPCR and normalized to β-actin, (E) The Bax/Bcl-2 ratio (n=3).

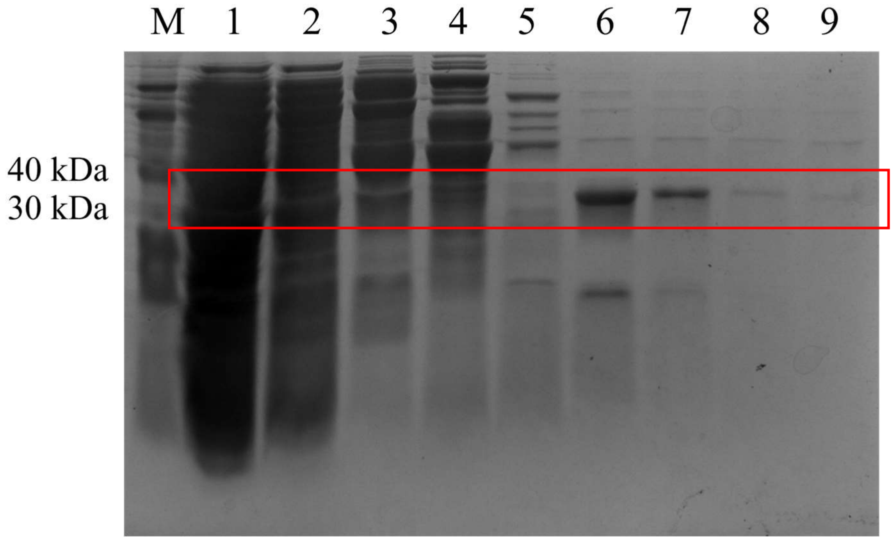

Figure S5. SDS-PAGE analysis of scFv T1 purification. M: Marker; 1: The sample after the expression bacteria have been lysed; 2: Flow-through liquid sample; 3: Balanced solution sample; 4: Buffer solution containing 40 mM imidazole; 5: Elution solution containing 100 mM imidazole; 6-8: Elution solution containing 200 mM

imidazole; 9: Elution solution containing 500 mM imidazole

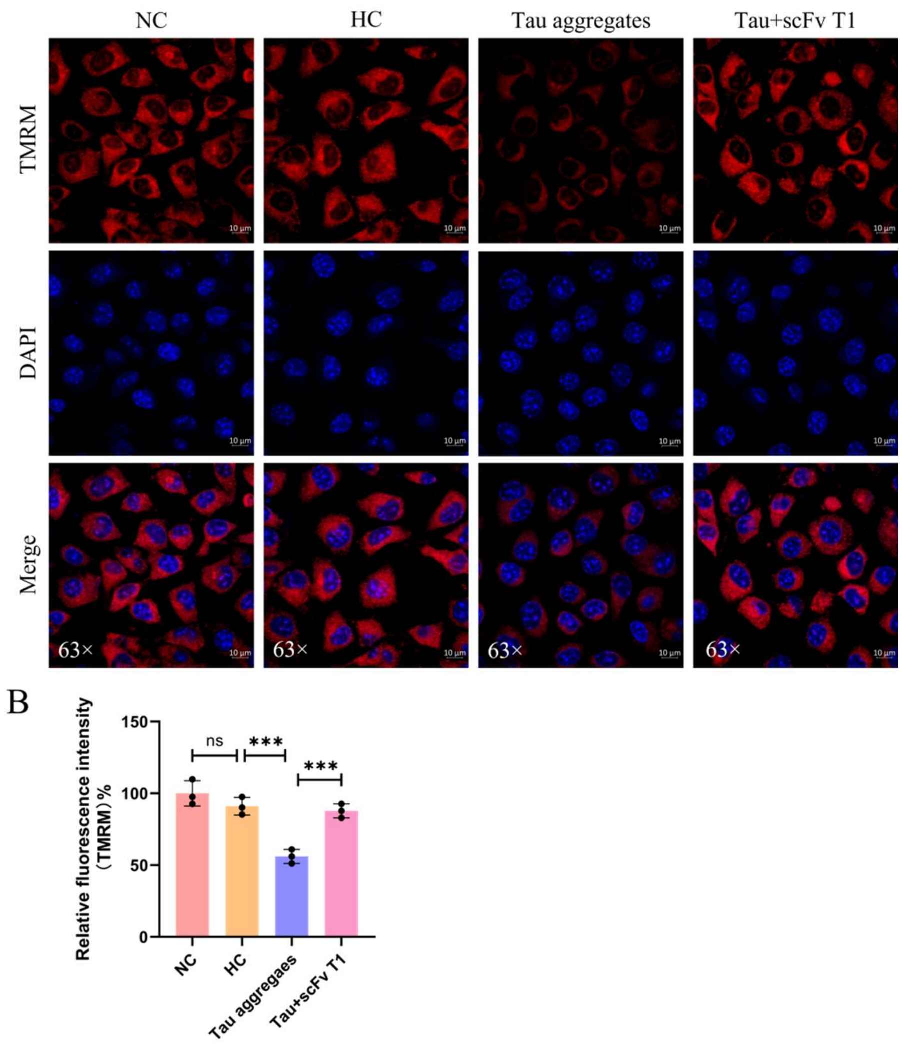

Figure S6. Mitochondrial membrane potential indicated by TMRM fluorescence. (A) TMRM staining. Scale bar, 5 μm. (B) Relative fluorescence intensity (TMRM) % of the cells in each group.
